# A 115-bp MethyLight assay for detection of *p16 *(CDKN2A) methylation as a diagnostic biomarker in human tissues

**DOI:** 10.1186/1471-2350-12-67

**Published:** 2011-05-13

**Authors:** Jing Zhou, Jie Cao, Zheming Lu, Hongwei Liu, Dajun Deng

**Affiliations:** 1 Key Laboratory of Carcinogenesis and Translational Research (Ministry of Education), Department of Aetiology, Peking University Cancer Hospital & Institute, Beijing, 100142, China; 2 Department of Oral Medicine, Peking University School of Stomatology, Beijing 100081, China

## Abstract

**Background:**

*p16 *Methylation is a potential biomarker for prediction of malignant transformation of epithelial dysplasia. A probe-based, quantitative, methylation-specific PCR (MSP) called MethyLight may become an eligible method for detecting this marker clinically. We studied oral mucosa biopsies with epithelial dysplasia from 78 patients enrolled in a published 4-years' followup cohort, in which cancer risk for patients with *p16 *methylation-positive dysplasia was significantly higher than those without *p16 *methylation (by 150-bp MSP and bisulfite sequencing; +133 ~ +283, transcription starting site, +1). The *p16 *methylation status in samples (*N *= 102) containing sufficient DNA was analyzed by the 70-bp classic (+238 ~ +307) and 115-bp novel (+157 ~ +272) MethyLight assays, respectively.

**Results:**

*p16 *Methylation was detectable in 75 samples using the classic MethyLight assay. The methylated-*p16 *positive rate and proportion of methylated-*p16 *by the MethyLight in MSP-positive samples were higher than those in MSP-negative samples (positive rate: 37/44 vs. 38/58, *P*=0.035, two-sided; proportion [median]: 0.78 vs. 0.02, *P <*0.007). Using the published results of MSP as a golden standard, we found sensitivity, specificity, and accuracy for this MethyLight assay to be 70.5%, 84.5%, and 55.0%, respectively. Because amplicon of the classic MethyLight procedure only partially overlapped with the MSP amplicon, we further designed a 115-bp novel MethyLight assay in which the amplicon on the sense-strand fully overlapped with the MSP amplicon on the antisense-strand. Using the 115-bp MethyLight assay, we observed methylated-*p16 *in 26 of 44 MSP-positive samples and 2 of 58 MSP-negative ones (*P *= 0.000). These results were confirmed with clone sequencing. Sensitivity, specificity, and accuracy using the 115-bp MethyLight assay were 59.1%, 98.3%, and 57.4%, respectively. Significant differences in the oral cancer rate were observed during the followup between patients (≥60 years) with and without methylated-*p16 *as detected by the 115-bp MethyLight assay (6/8 vs. 6/22, P = 0.034, two-sided).

**Conclusions:**

The 115-bp MethyLight assay is a useful and practical assay with very high specificity for the detection of *p16 *methylation clinically.

## Background

Aberrant methylation of CpG islands is a very stable modification of genomic DNA that often inactivates gene expression pathologically. Methylation of a target CpG island in even 0.1% of a cell population obtained from fixed/frozen tissues or body fluids can be detected readily. The high stability and high sensitivity of detection make DNA methylation one kind of optimal clinical biomarker for the prediction of potential malignancy progression of precancerous lesions, metastasis/recurrence of cancer, and chemo/radio-therapy sensitivity [1].

It is well recognized that complete methylation of CpG sites within CpG islands around transcription start sites represents deep-silencing of gene expression established during embryo development and cell differentiation. Well-documented examples include the silencing of tissue-specific genes, gene imprinting, inactivation of parasite DNA and X-chromosome. However, the methylation of CpG islands in tumor suppressor genes, including *p16*, is a progressive process encountered during carcinogenesis [2-4]. *De novo *methylation often occurs post gene silencing at a few seeding CpG sites in initiation and precancerous stages, and ultimately extends to the full CpG island in advanced cancer. This complicates the development of an assay to detect the methylation status of a target CpG island in which complete methylation is not established. For example, methylation of crucial CpG sites within a CpG island that correlates with clinical outcomes should first be identified, and then a proper detection approach with high specificity for clinical diagnosis should be designed. Unfortunately, such crucial CpG sites are not well characterized for most CpG islands. This often leads to the dissimilar detection of methylation at different CpG sites within a target CpG island between different laboratories. Contradictory results often arise from different kinds of detection assays, or the same assay with different detection sensitivity [5].

**Figure 1:**
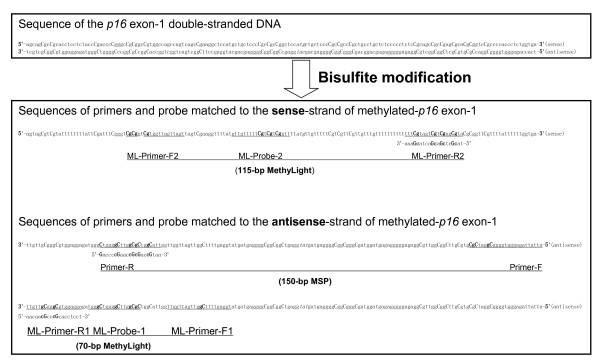
**The sequence of the *p16 *exon-1 before and after bisulfite conversion**. Locations of amplicons, primers, and probes used in the 150/151-bp MSP-m/u, 70-bp, and 115-bp MethyLight assays within the sense-strand or antisense-strand were underlined and labelled.

Tumor suppressor gene *p16 *(CDKN2A) controls cell proliferation through the P16-CDK4-RB pathway at the G1→S checkpoint of the cell cycle [6]. Frequent, aberrant methylation of a crucial CpG island is the main mechanism of inactivation for *p16 *in the early stages of carcinogenesis [1]. A number of nested case-control studies and followup cohorts consistently showed *p16 *methylation as a potential biomarker for the early prediction of malignant transformation of epithelial dysplasia, one kind of precancerous lesion in many organs/tissues including the oral/oesophageal/gastric mucosa [7-13]. Although bisulfite-clone sequencing provides detailed information about the methylation status of each CpG site in the cloning molecules, it is often used as a confirmation assay rather than a regular detection assay because of its low detection sensitivity (> 20%), labor, and time costs. A number of assays including MSP, MethyLight, Pyrosequencing, and DHPLC are often used to detect *p16 *methylation in laboratory research [3,7-16]. Among them, MethyLight, based on MSP primers, may become one of the most eligible, convenient, quantitative, and sensitive assays for the clinical detection of *p16 *methylation primarily because it uses a methylation-specific primer set and real-time, sequence-specific probe validation. In the present study, we evaluated the sensitivity, specificity, and accuracy of a 70-bp classic assay in which the amplicon partially overlapped with the MSP amplicon, and a 115-bp novel MethyLight assay in which the amplicon fully overlapped with the MSP amplicon (Figure 1). The data was collected from 102 oral epithelial dysplasia samples obtained from a followup cohort study, in which malignant transformation of this disease correlated with *p16 *methylation detected by MSP and was confirmed by clone sequencing [13].

## Results and Discussion

### Detection of *p16 *methylation by a classic 70-bp MethyLight assay

An eligible PCR-based molecular assay for diagnosis should meet several essential requirements including high specificity, real-time validation using a sequence-specific probe, positive confirmation with direct sequencing, and refractory to carry-over contamination. Combination of MethyLight using methylation-specific primers with probes containing an anti-contamination system, composed replacing dTTP with dUTP and the addition of a uracil glycosylase UNG in the PCR reaction mixture, may become an ideal method for the clinical detection of methylation in a specific CpG island. In a 4-year followup cohort, we reported that methylated-*p16 *was a potential biomarker for early prediction of malignant transformation of oral epithelial dysplasia [13]. Among patients of at least 60 years of age, the sensitivity and specificity of methylated-*p16 *were 77% and 78%, respectively. Hall *et al. *reported similar results [14]. Therefore, the using MethyLight as a clinical assay to detect methylated-*p16 *was feasible.

**Figure 2:**
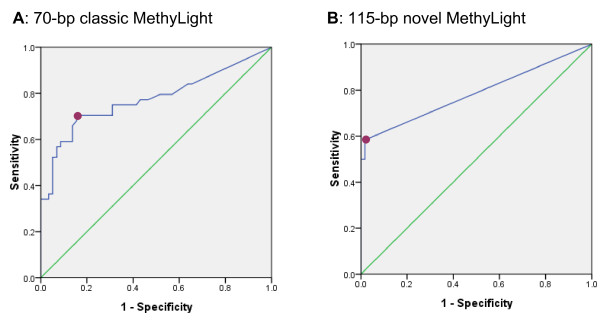
**ROC curves of detection of methylated-*p16 *by two MethyLight assays**. The sensitivity and specificity of the MethyLight assay at various points of relative copy number in 102 tested samples were calculated according to the result of the 150-bp MSP. The cut-off points of RCN were marked by the deep-red circlets. **A**: For the 70-bp classic MethyLight, the area under the curve is 0.776 (95% CI: 0.677-0.874), P = 0.000. When the cut-off point of RCN was 0.0725, the sensitivity and specificity were 0.705 and 0.845, respectively. **B**: For the 115-bp novel MethyLight assay, the area under the curve is 0.787 (95% CI: 0.689-0.884), P = 0.000. When the cut-off point of RCN was 0.0002, the sensitivity and specificity were 0.591 and 0.983, respectively.

The 70-bp classic MethyLight for methylated-*p16 *was evaluated using either genomic DNA of baseline or followup samples (*n *= 102) from patients enrolled in the mentioned cohort (*n *= 78). After genomic DNA was converted to SafeBis templates as described in the methods section, the methylated-*p16 *was analyzed with the classic MethyLight. Methylated-*p16 *was detected in 75 of 102 tested samples. The methylated-*p16 *MethyLight-positive rate and proportion of methylated-*p16 *in 44 methylated-*p16 *MSP-positive samples were higher than those in 58 MSP-negative samples, respectively (positive rate: 37/44 vs. 38/58, *P *= 0.035, two-sided; proportion [*median*]: 0.78 vs. 0.02, *P <*0.007). Using the prognosis-related MSP-results of methylated-*p16 *as a golden standard, we found sensitivity, specificity, and accuracy for the classic MethyLight were 70.5%, 84.5%, and 55.0% with a cut-off point of RCN set at 0.073, respectively (Figure 2A).

### Development of a 115-bp novel MethyLight assay

After conversion of unmethylated cytosine residues to uracil (or thymine in PCR products; C → U/T) residues, a double stranded DNA molecule is transformed into two non-complementary single-stranded DNA molecules (C≡G → U/T≠G), as illustrated in Figure 1. Interestingly, all current methylation detection assays for the *p16 *CpG islands are designed according to the antisense-strand sequence of the *p16 *exon-1, while none target the sense-strand. The main reasons may include the good performance of first 150/151-bp MSP-m/u for methylated/unmethylated-*p16 *in cell line and tissue samples, and the very high content (111/175) of thymine residues in the unmethylated sense-strand present after bisulfite modification, which makes it difficult to design a proper unmethylation-specific primer set that can be used as control MSP-u in the case that *p-16 *is not methylated (Figure 1). However, in the MethyLight assay, instead of using the template corresponding to unmethylated *p-16, *the *COL2A1 *gene, without a CpG island, is recommended as an optimal common reference for all tested CpG islands for quantification of modified genomic DNA in the tested samples [17]. Using this strategy, the sense-strand of the methylated-*p16 *can be used to design a MethyLight assay.

**Figure 3:**
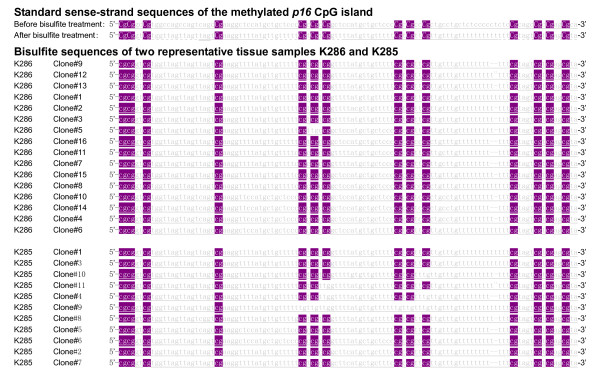
**Results of clone sequencing of the 115-bp MethyLight PCR products**. The sense-strand sequences of the methylated *p16 *CpG island with and without bisulfite treatment were listed. The bisulfite-treated template of two 115-bp MethyLight-positive samples was amplified with the same primer set. The PCR products of these two representative samples were clone-sequenced, respectively. 99.6% (223/224) and 93.5% (144/154) cytosines at CpG sites within total 16 and 11 clones (14 CpG sites/clone) from the sample K286 and K285 were maintained and 77.8% (548/704) and 90.9% (440/484) cytosines at non-CpG sites within these clones (44 non-CpG sites/clone) were converted to thymines, respectively. These results indicate that these clones are fully methylated at all CpG sites.

The amplicon (+238 ~ +307; transcription starting site, +1) of the 70-bp MethyLight partially overlapped with the 150-bp MSP amplicon (+133 ~ +283) (Figure 1). To investigate the feasibility of using the *p16 *exon-1 sense-strand for detection of methylated-*p16*, we designed a 115-bp novel MethyLight assay according to the sense-strand believing it might correlate with the 150-bp MSP better than the 70-bp MethyLight assay. This theory was based on the fact that the 115-bp MethyLight amplicon matched to the 150-bp MSP amplicon better than the 70-bp MethyLight amplicon (Figure 1). Using the novel MethyLight assay, we observed methylated-*p16 *in 26 of 44 MSP-positive samples and 2 of 58 MSP-negative ones (*P*=0.000). This result was confirmed by clone sequencing two representative samples (Figure 3). When the RCN cut-off point was set at 0.0002, the sensitivity, specificity, and accuracy of the 115-bp MethyLight were 59.1%, 98.3%, and 57.4%, respectively (Figure 2B).

### Comparison of two MethyLight assays

**Figure 4:**
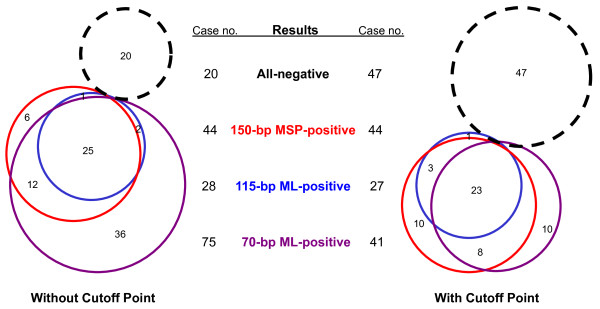
**Overlapping information of methylated-*p16 *by different assays**. 102 oral mucosa biopsy samples were tested using three assays; red circles indicate methylated-*p16 *positive sample numbers as determined using the 150-bp MSP; blue circles indicate methylated-*p16 *positive sample numbers as determined using the 115-bp novel MethyLight assay; violet circles indicate methylated-*p16 *positive sample numbers as determined using the 70-bp classic MethyLight assay; the black dashed line circles represent samples without methylated-*p16 *detected using the three assays; the number within each open area covered by different cycles represents the exact number of samples containing methylated-*p16 *as detected by the corresponding assays. **A**: Qualitative results without the use of a cut-off point. **B**: Summary of the total number of samples with or without methylated-*p16 *as determined by each assay (left/right case no., without/with cut-off point). **C**: Qualitative results using a cut-off point for the two MethyLight assays (relative copy number; 0.073 for the classic MethyLight assay, and 0.0002 for the novel MethyLight assay).

Furthermore, we compared the results of two MethyLight assays and found 23 of the 41 (56.1%) classic MethyLight positive samples are also 115-bp MethyLight positive; whereas only 4 of 61 (6.6%) classic MethyLight negative samples are 115-bp MethyLight positive (P = 0.000). The detailed overlap information for the results of methylated-*p16 *in all 102 tested samples was analyzed (Figure 4). Apparently, when the RCN cut-off points were set for the two MethyLight assays, the novel and classic assays had the similar accuracy (57% and 55%). However, the 115-bp MethyLight had a very high specificity, while the classic MethyLight had a higher sensitivity. Most importantly, the sensitivity and specificity of the novel MethyLight assay are consistent regardless of whether the RCN cut-off point was used during the calculation process. This indicates the 115-bp assay could be used as a qualitative assay for clinical detection of methylated-*p16*.

We further analyzed the clinical outcome of methylated-*p16 *as detected by two MethyLight assays. Among 30 patients of at least 60 years of age, methylated-*p16 *was detected in 8 baseline samples by the 115-bp MethyLight assay (with or without the cut-off value). During the followup period, oral cancer developed in 6 of 8 methylated-*p16 *positive patients (75.0%), but only 6 of 22 patients (27.3%) without methylated-*p16 *developed oral cancer [odd ratio 8.00 (95% CI, 0.98~80.93; P = 0.034, two-sided). Among 34 patients analyzed using the classic MethyLight assay (with cut-off value 0.073), the odds ratio of methylated-*p16 *was 3.64 (6/10 vs. 7/24; 95% CI, 0.62~21.91; P = 0.130). These results suggest that the 115-bp MethyLight assay might be better suited to detect the methylated-*p16 *biomarker than the classic MethyLight assay.

## Conclusions

The 115-bp MethyLight assay maybe a practical assay for the detection of methylated-*p16 *biomarker for clinical diagnosis.

## Methods

### Patients and oral biopsies

102 genomic DNA samples (> 500 ng) were extracted from paraffin-embedded oral mucosa biopsies containing mild or moderate dysplasia lesions from 78 patients enrolled in a 4-year follow-up cohort (NCT00835341, available at http://ClinicalTrials.gov) [7,13]. Briefly, the fixed tissue block was cut into 10 μm slides, treated with xylene to remove the paraffin, rehydrated with graded ethanol, mixed with lysis buffer containing 100 μg proteinase K, digested at 56°C overnight, and incubated 10 min at 95°C to stop the digestion [18]. DNA present in the digestion solution was precipitated with ethanol and dissolved in 50 μl TE buffer. DNA concentration was determined spectrophotometrically with diphenylamine as described [19]. The average recovery rate of genomic DNA was 77.6%. 61 samples were baseline biopsies and the remaining 41 samples were taken during the followup periods. Methylation status of the antisense-strand of exon-1 within the *p16 *CpG island was determined using a 150-bp MSP assay in which DHPLC was used as the detector; the results were further confirmed through clone sequencing (Figure 1). Methylated-*p16 *was detected in 44 of these samples. The study was approved by the Institutional Review Boards of Peking University School of Stomatology and School of Oncology, and all patients gave written informed consent.

### Preparation of SafeBis DNA by bisulfite treatment

Genomic DNA samples (2 μg) were treated with bisulfite for 16 hrs at 50°C without desulfonation as described [20], purified with the Wizard DNA Clean-Up System Kit (Promega, Madison, WI), dissolved in 40 μl TE preheated to 80°C, and stored in three aliquots at -20°C before use. The unmethylated cytosine residues in the DNA were converted to uracil (thymine in PCR products) and the methylated cytosine residues remained intact after this treatment.

### Detection of *p16 *methylation by the 70-bp classic MethyLight assay

Methylation of CpG sites across the MSP Primer-R region in the antisense-strand of the *p16 *exon-1 was analyzed by the classic MethyLight assay using modified primers [15]. Briefly, the ML-Primer-F1 (5'-tgga*g *tttt**C g**gttg attgg tt-3'), ML-Primer-R1 (5'-aacaa c**G*c***cc **Gc**acc tcct-3'), and a methylated-*p16*-specific ML-Probe-1 (6FAM5'-acc**Cg **accc**C g**aac**C gCg**-3'TAMRA, TaqMan) were used to detect the 70-bp methylated *p16 *templates in the SafeBis DNA (Figure 1). The reference gene *COL2A1 *was also amplified with a forward primer (5'-tctaa caatt ataaa ctcca accac caa-3'), a reverse primer (5'-gggaa gatgg gatag aaggg aatat-3'), and a *COL2A1*-specific probe (6FAM5'-ccttc attct aaccc aatac ctatc ccacc tctaa a-3'BHQ1) [17]. A uracil DNA glycosylase (UNG) carry-over prevention system was employed in the MethyLight assay [18]. The 20 μl MethyLight reaction mixture contained 2 μl 10×PCR buffer (Qiagen, Germany), 0.5 units of HotStar Taq DNA polymerase (Qiagen), 200 μmol/L dATP, 200 μmol/L dCTP, 200 μmol/L dGTP, 800 μmol/L dUTP (Promaga), 5 mmol/L MgCl_2_, 75 nmol/L of each primer (TaKaRa, Beijing), 75 nmol/L probe (TaKaRa), 2 μl 10×UNG Buffer (NEB), 0.4 units UNG (NEB), and 10 ng template. An ABI7500 thermal cycler was used to conduct the PCR reactions using the following thermal conditions: 37°C for 10 min → 95°C for 30 min → (95°C for 15 sec → 62°C for 1 min) × 45 cycles. The fluorescence value was detected at 62°C. Duplicate tubes were used for each sample, and the average Ct value was used in the calculations. Relative copy number (RCN) of methylated-*p16 *was calculated according to the formula [2^-ΔCt^, (ΔCt = Ct_methylated__*-p16 *_- Ct_*COL2A1*_)]. RKO and MGC803 xenografts from nude mice were also used as methylated-*p16 *positive and negative controls in each experiment, respectively [13]. The calculated RCN of methylated-*p16 *in each sample was standardized according to the RCN of RKO positive control.

### Detection of *p16 *methylation by the 115-bp MethyLight assay

The ML-Primer-F2 (5'-**CgCg**g t**Cg**tg gttag ttagt-3'), ML-Primer-R2 (5'-ta**cG**c t**cG**a**c G**acta **Cg**aaa-3'), and ML-Probe-2 (5'-6FAM-gttgt tttt**C g**t**Cg**t **Cg**gtt-TAMRA-3') were used to detect the 115-bp methylated fragment of the sense-strand of *p16 *exon-1, which completely overlapped the sense-strand sequence corresponding to the 150-bp MSP amplicon within the antisense-strand (Figure 1). Other conditions were the same as the classic MethyLight assay.

### Clone sequencing of the 115-bp MethyLight PCR products of methylated-*p16*

The SafeBis template from two representative samples of the 115-bp MethyLight-positive samples was amplified with the same primer set used in the 115-bp MethyLight assay (without the ML-Probe-2), and then clone-sequenced as described [3].

### Statistical methods

A ROC curve of the results for each MethyLight assay was calculated. Results of methylated-*p16 *in these tested samples, determined using the 150-bp MSP-m (and by151-bp MSP-u in the MSP-m negative cases), were used as the golden standard in the calculation of sensitivity and specificity for the two MethyLight assays (Figure 1). These results showed a strong correlation with the malignant transformation of these lesions in the 4-year followup cohort study [13]. The accuracy was calculated according to the formula [Sensitivity+Specificity-1]. The Chi-square test and Student's t-test were used to test the significance of qualitative and quantitative data between different groups. All tests were two-sided.

## Authors' contributions

JZ carried out the molecular epigenetic assays. ZL carried out the clone sequencing. JC and HL collected the tested samples. DD conceived the study and drafted the manuscript. All authors read and approved the final manuscript.

## Pre-publication history

The pre-publication history for this paper can be accessed here:

http://www.biomedcentral.com/1471-2350/12/67/prepub
